# Supplementary material for: Photoreactive Coating Material as an Effective and Durable Antimicrobial Composite in Reducing Bacterial Load on Surfaces in Livestock
Source: Biomedicines. 2022 Sep 17;10(9):2312. doi: 10.3390/biomedicines10092312 (PMC9496029; doi:10.3390/biomedicines10092312)
Supplement: Supplementary file 1 [file biomedicines-10-02312-s001.zip › biomedicines-1885492-supplementary.pdf]

Supplemental material

Photoreactive coating material as an effective and durable antimicrobial composite in reducing bacterial load on surfaces in livestock

Ádám Kerek [1, *], Mátyás Sasvári [1], Ákos Jerzsele [1], Zoltán Somogyi [1], László Janovák [2], Zsolt Abonyi-Tóth [3] and Imre Dékány [2]

1 Department of Pharmacology and Toxicology, University of Veterinary Medicine, István Street 2, H-1078 Budapest, Hungary
2 Department of Physical Chemistry and Materials Science, University of Szeged, Rerrich Béla tér 1, H-6720 Szeged, Hungary
3 Department of Biomathematics and Informatics, University of Veterinary Medicine, István Street 2, H-1078 Budapest, Hungary
* Correspondence: kerek.adam@univet.hu

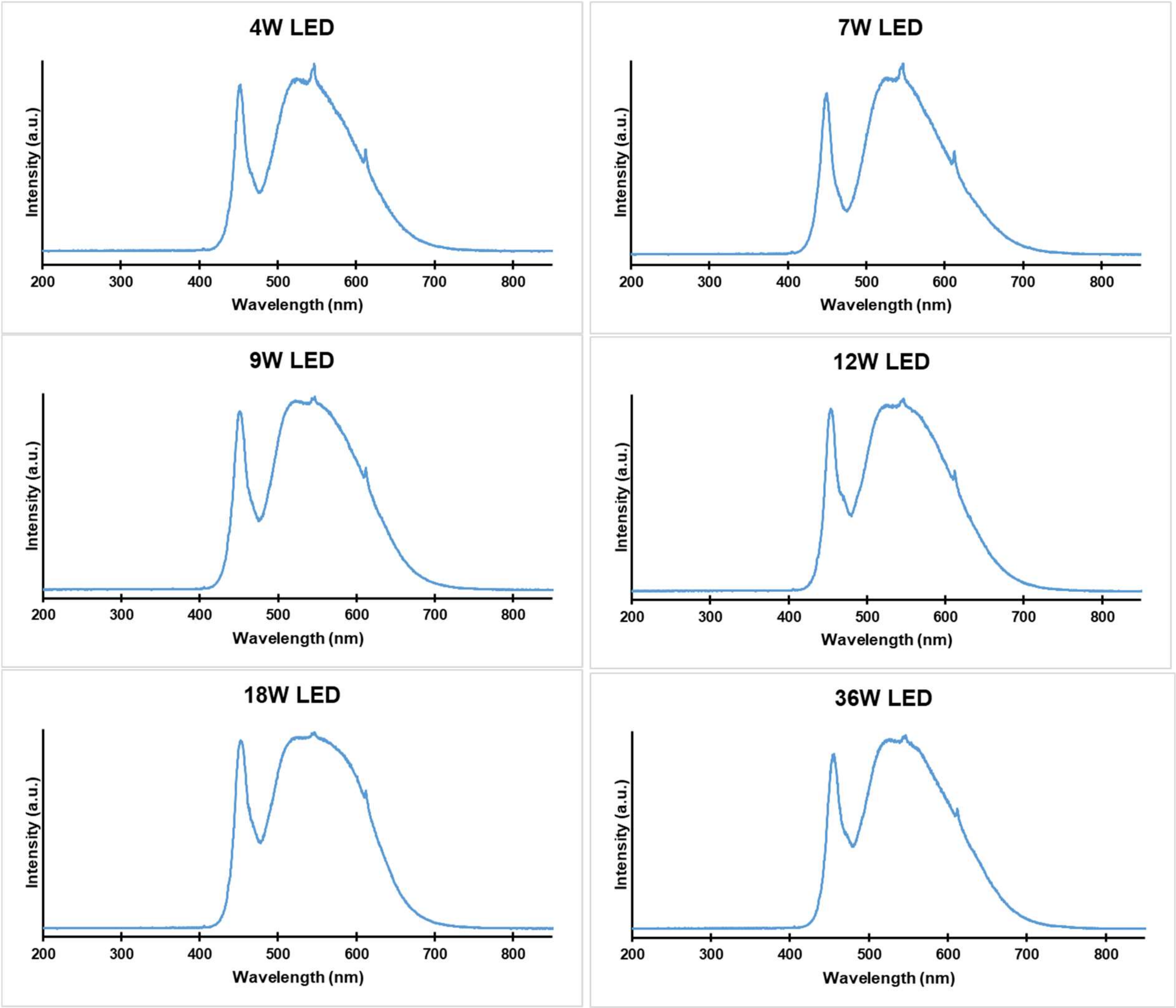

Figure S1. The emission spectra of LED light sources with increasing wattage.

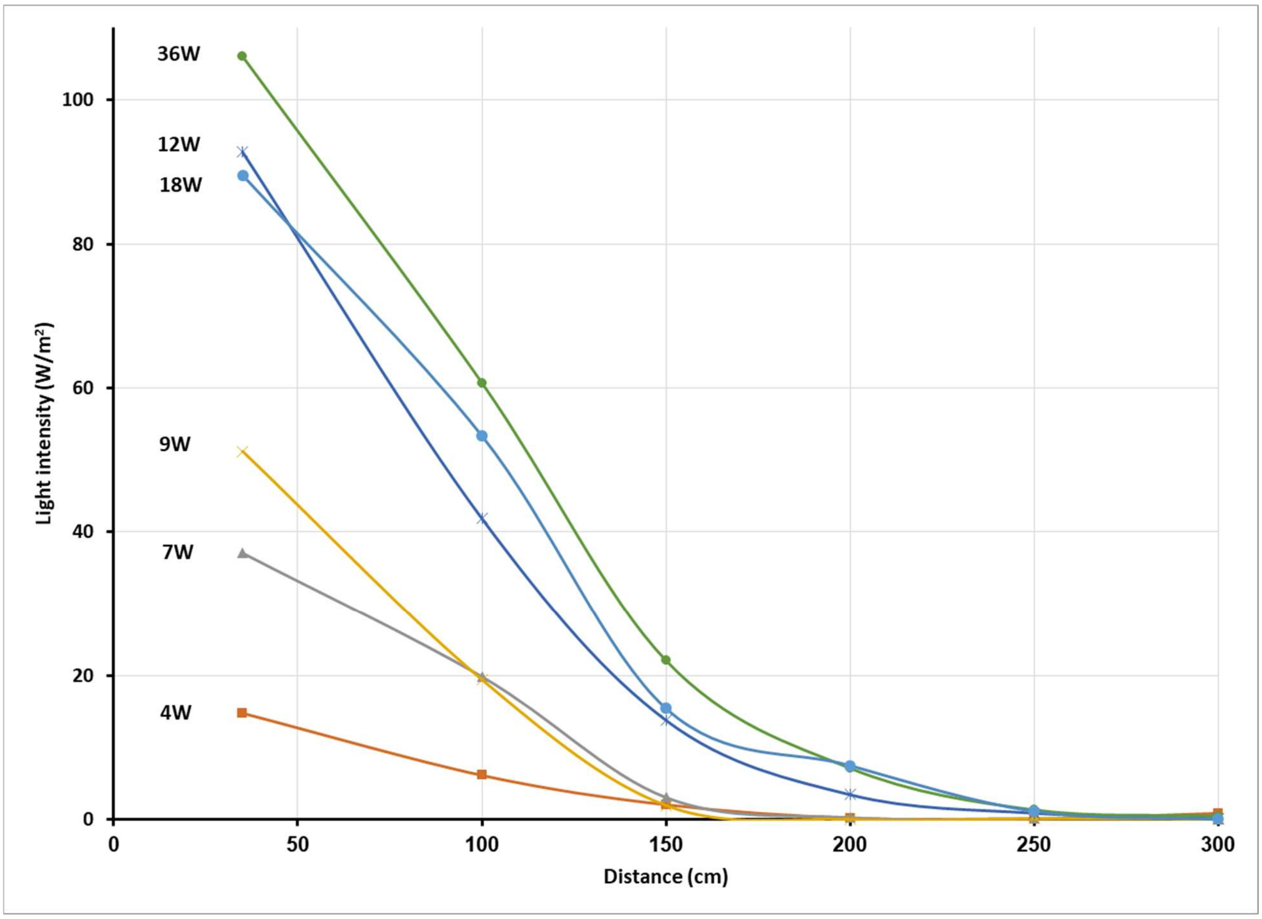

Figure S2. The measured light intensity values of LED light sources as a function of the distance
